# Comparing the effects of insulin glargine and thiazolidinediones on plasma lipids in type 2 diabetes: a patient-level pooled analysis

**DOI:** 10.1002/dmrr.1305

**Published:** 2012-03

**Authors:** Ajay Chaudhuri, Julio Rosenstock, Andres DiGenio, Luigi Meneghini, Priscilla Hollander, Janet B McGill, Paresh Dandona, John Ilgenfritz, Matthew Riddle

**Affiliations:** 1 State University of New York at Buffalo and Kaleida HealthBuffalo, NY, USA; 2 Dallas Diabetes and Endocrine Center at Medical CityDallas, TX, USA; 3 Sanofi-aventis USBridgewater, NJ, USA; 4 University of Miami Miller School of MedicineMiami, FL, USA; 5 Baylor University Medical CenterDallas, TX, USA; 6 Washington University in St. Louis School of MedicineSt. Louis, MO, USA; 7 United Biosource CorporationBlue Bell, PA, USA; 8 Oregon Health & Science UniversityPortland, OR, USA

**Keywords:** insulin glargine, lipids, thiazolidinediones, type 2 diabetes

## Abstract

**Background:**

The prevalence of dyslipidaemia and the risk of cardiovascular disease are elevated in patients with type 2 diabetes. This analysis compared the effects of insulin glargine *versus* thiazolidinediones (TZDs) on lipid profiles.

**Methods:**

Patient-level data were pooled from two randomized clinical studies. The population included 552 men and women aged >18 years, diagnosed with type 2 diabetes for at least 6 months, on metformin and/or sulphonylurea, and with A_1C_ ≥7.5% and <12.0% at screening. Lipid outcome measures included change from baseline in lipid levels [low-density lipoprotein cholesterol (LDL-C), high-density lipoprotein cholesterol (HDL-C), non-high-density lipoprotein cholesterol (non-HDL-C), total cholesterol, triglycerides, and free fatty acids] and attainment of lipid goals for LDL-C, non-HDL-C, and triglycerides.

**Results:**

Both insulin glargine and TZDs improved lipid profiles from baseline values. Compared with TZDs, treatment with insulin glargine led to 7.9% greater reduction in LDL-C (*p* < 0.0003), 7.5% greater reduction in non-HDL-C (*p* < 0.0001), and 7.8% greater reduction in total cholesterol (*p* < 0.0001), whereas the HDL-C increase with TZD was 7.6% greater than that with insulin glargine (*p* < 0.0001). The percentage of patients attaining the lipid goals was comparable between insulin glargine and pioglitazone, but lower for rosiglitazone. Insulin glargine improved glycaemic control more than TZDs; however, insulin glargine caused more hypoglycaemia. Treatment with TZDs caused more weight gain and peripheral oedema.

**Conclusion:**

These findings suggest that the favourable effects of insulin glargine on plasma lipid profiles should be considered among the advantages of treatment with insulin glargine as they are for TZDs. Copyright © 2011 John Wiley & Sons, Ltd.

## Introduction

It is well established that the prevalence of dyslipidaemia and the risk of cardiovascular disease (CVD) are greater in patients with type 2 diabetes than in the general population [1–4]. Elevated plasma lipid concentrations are well-recognized risk factors for CVD, and reducing them to the values recommended by the American Diabetes Association (ADA) and the American Heart Association (AHA) is a primary strategy in CVD prevention in individuals with type 2 diabetes [1,5–7]. Thus, the effect of antihyperglycaemic therapies on lipid parameters merits consideration in the treatment of type 2 diabetes patients.

Over the years, insulin was suspected to be atherogenic [8–10], whereas thiazolidinediones (TZDs) were considered to have cardioprotective potential [11,12]. These properties have been regarded to contribute to the unfavourable effects of insulin and beneficial effects of TZDs on lipids [13,14]. However, clinical studies indicate that insulin therapy produces favourable effects on plasma lipid concentrations in patients with type 2 diabetes [15–18]. In particular, treatment with insulin glargine has been associated with improvement in the levels of low-density lipoprotein cholesterol (LDL-C), high-density lipoprotein cholesterol (HDL-C), non-high-density lipoprotein cholesterol (non-HDL-C), total cholesterol (TC), and triglycerides (TGs) [15,16,18]. Therefore, to investigate in greater detail the comparative effects of insulin glargine, rosiglitazone, and pioglitazone on lipoprotein and glycaemic parameters, we identified two randomized clinical studies that compared insulin glargine with a TZD in the treatment of type 2 diabetes [19,20]. Although these studies were designed primarily to evaluate glycaemic control, lipoprotein levels were also determined [19,20]. One study compared insulin glargine with rosiglitazone [19], and another study compared insulin glargine with pioglitazone [20]. The pooled analysis suggests that insulin glargine has positive effects on several lipoprotein parameters often seen elevated in this population, and confirms the known effects of TZDs on lipids [19,20].

## Materials and methods

Participant-level data were pooled from two previously published randomized studies comparing the efficacy, safety, and tolerability of insulin glargine *versus* a TZD, either pioglitazone or rosiglitazone, in type 2 diabetes. These studies [19,20] were selected for analysis because they had similar patient profiles, included thorough measurement of lipid parameters, and compared the effects of insulin glargine with one of the two TZDs available at the time of the study. Each study used a randomized, parallel-group, two-arm, open-label design with outcome measurements after 24 weeks of treatment. The participants included men and women aged >18 years, diagnosed with type 2 diabetes for at least 6 months, on metformin and/or sulphonylurea, and with A_1C_ ≥7.5% and <12.0% at screening.

The participants in the analysis were randomized patients who had baseline measurements, received at least one dose of study medication, and had outcome measurements from at least one follow-up visit. Patient-level data from the 552 participants who met these criteria were included in the pooled analysis.

### Treatments

Patients were randomized to insulin glargine titrated to a fasting plasma glucose (FPG) goal of <5.55 mmol/L (<100 mg/dL) or to a TZD comparator level (pioglitazone or rosiglitazone) [19,20]. Detailed information regarding treatment procedure is available in prior publications [19,20]. Study protocols were approved by the respective independent review boards, and both studies were conducted in accordance with the principles of the Declaration of Helsinki. Written informed consent was obtained from study participants prior to any treatment initiation.

Because of the design of the individual studies, all patients randomized to rosiglitazone also were taking both metformin and a sulphonylurea [19], and all patients receiving pioglitazone were taking either metformin or a sulphonylurea [20]. For patients who were taking a lipid-lowering medication (e.g. statin or fibrate) at study onset, titration of these medications was permitted under the protocol at the discretion of the treating physician.

### Outcome measures

The lipid outcome measures for this post hoc analysis were (1) change from baseline in plasma lipid levels: LDL-C, HDL-C, non-HDL-C, TC, TGs, and free fatty acids (FFAs); (2) attainment of ADA/AHA recommended goals for LDL-C [<2.59 mmol/L (<100 mg/dL)], non-HDL-C [<3.37 mmol/L (<130 mg/dL)], and TGs [<1.70 mmol/L (<150 mg/dL)]; and (3) attainment of TC/HDL-C <4.5 and LDL-C/HDL-C <3.5. Glycaemic control was measured by change from baseline in A_1C_ and FPG and proportion of patients achieving A_1C_ ≤7.0%. The safety evaluation included body weight, peripheral oedema, and episodes of symptomatic and severe hypoglycaemia.

Fasting blood samples were collected at baseline and weeks 6, 12, 18, and 24 and were sent for analysis to a central laboratory that participated in the Lipid Standardization Program of the Centers for Disease Control (Covance Central Laboratory Services, Indianapolis, IN). The TC and TG assays were performed using Hitachi analyzers (Hitachi, Ltd, Japan). Direct HDL-C was measured with an enzymatic colorimetric assay (Roche Diagnostics, Indianapolis, IN). Low-density lipoprotein cholesterol was calculated using the Friedewald formula (TC − HDL-C − TG/5) [21]. FFAs were measured using the Wako enzymatic non-esterified (free) fatty acids (NEFA) method. The A_1C_ assay was performed on a Bio-Rad Variant™ Analyzer (Bio-Rad Laboratories, Hercules, CA).

### Statistical analysis

Analysis of covariance (ANCOVA) was used to evaluate change from baseline for lipid outcomes, glycaemic outcomes, and weight. Each ANCOVA model included treatment (insulin glargine *versus* pooled TZD) and study as fixed effects and corresponding baseline level as a covariate. To account for the use of lipid-lowering medications, additional ANCOVAs were conducted for change from baseline in each lipid outcome, with treatment (insulin glargine *versus* pooled TZD) and statin/fibrate use (yes *versus* no) as fixed effects and lipid baseline level as covariate. Lipid parameters were analysed on a log scale, to adjust for non-normal distributions, and then back-transformed for reporting results. If measurement of an outcome variable was not available at week 24, then the last observation post-baseline was carried forward.

Continuous lipid values measured at baseline were categorized into quartiles. Analysis of variance of change from baseline was conducted for each lipid outcome variable, with treatment and baseline quartile as factors. To compare the effects of the three treatments (insulin glargine, pioglitazone, and rosiglitazone), paired comparisons on the change from baseline lipid concentration were performed using *t*-tests.

Stepwise linear regression models were used to identify additional factors that may have contributed significantly to lipid outcomes in this analysis. For each lipid parameter, 26 potential covariates were available for selection: age, sex, race, body mass index (BMI), duration of diabetes, treatment (insulin glargine or TZD), study (Rosenstock or Meneghini), concomitant statin/fibrate use, concomitant use of blood pressure medication, background use of sulphonylurea, background use of metformin, background use of metformin and sulphonylurea, baseline A_1C_, baseline FPG, baseline weight, baseline systolic blood pressure, baseline diastolic blood pressure, baseline heart rate, baseline TC, baseline LDL-C, baseline HDL-C, baseline TGs, baseline FFA, change in FPG from baseline, change in A_1C_ from baseline, and change in weight from baseline. A significance level of *p* ≤ 0.15 was required for a covariate to enter into the model and *p* ≤ 0.10 to be retained in the final model.

## Results

Table 1 shows the demographic and baseline characteristics of the 552 patients included in the pooled analysis. A total of 264 patients were randomized to insulin glargine and 288 patients to TZD (112 to rosiglitazone and 176 to pioglitazone). Not all patients had complete lipid data, so analyses of most lipid outcomes included 258 patients treated with insulin glargine and 278 with TZD (110 rosiglitazone and 168 pioglitazone). Low-density lipoprotein cholesterol values were not calculated for 54 participants (24 insulin glargine, 9 rosiglitazone, 21 pioglitazone) with TGs >4.52 mmol/L (>400 mg/dL) because LDL-C calculation becomes unreliable under such conditions [21.

**Table 1:** Demographic and baseline characteristics

	Insulin glargine (*n* = 264)	All TZDs (*n* = 288)
Age (years)	54.2 ± 10.9	53.3 ± 10.7
Female, *n* (%)	134 (50.8)	134 (46.5)
Race, *n* (%)		
White	171 (64.8)	199 (69.1)
Black	51 (19.3)	44 (15.3)
Hispanic	32 (12.1)	39 (13.5)
Other	10 (3.8)	6 (2.1)
Duration of diabetes (years)	7.3 ± 5.5	6.8 ± 4.7
Lipid-reducing therapy, statin or fibrate, *n* (%)	103 (39.0)	107 (37.2)
Background oral anti-diabetic drug therapy, *n* (%)		
Metformin^a^	90 (34.1)	103 (35.8)
Sulphonylurea^a^	69 (26.1)	73 (25.3)
Metformin + sulphonylurea^a^	105 (39.8)	112 (38.9)
Weight (kg)	96.8 ± 20.5	97.9 ± 21.1
BMI (kg/m^2^)	34.1 ± 7.8	33.7 ± 6.6
Lipid values, mean ± SE (mg/dL)^b^		
LDL-C	109.2 ± 2.12	106.7 ± 2.21
HDL-C	42.3 ± 1.46	41.5 ± 1.44
Non-HDL-C	151.2 ± 1.74	150.5 ± 1.69
Total cholesterol	195.6 ± 1.39	194.2 ± 1.33
Triglycerides	194.3 ± 4.18	198.8 ± 3.63
Free fatty acids	52.9 ± 2.72	51.2 ± 2.50
Glycaemic values		
A_1C_ (%)	9.1 ± 1.1	9.1 ± 1.2
FPG (mg/dL)	209.1 ± 63.3	209.1 ± 61.3^c^

Data are mean ± SD, except where indicated.

BMI, body mass index; FPG, fasting plasma glucose; HDL-C, high-density lipoprotein cholesterol; LDL-C, low-density lipoprotein cholesterol; non-HDL-C, non-high-density lipoprotein cholesterol; TZD, thiazolidinedione.

a Because of differences in study design, all 176 TZD patients taking either metformin or a sulphonylurea were treated with pioglitazone, and all 112 TZD patients taking metformin and a sulphonylurea were treated with rosiglitazone.

b Lipid levels reported as geometric means.

c *n* = 287.

### Changes in plasma lipid levels

Changes in lipid parameters are shown in Table 2; adjusted values were controlled for study differences and baseline lipid concentrations. There were significant differences between the effects of insulin glargine and pooled TZD on the levels of LDL-C (*p* = 0.0003), non-HDL-C (*p* < 0.0001), and TC (*p* < 0.0001); insulin glargine treatment reduced whereas pooled TZD treatment increased these cholesterol levels. Insulin glargine therapy had favourable effects on TGs and FFAs, but the differences were of borderline statistical significance. For example, treatment with insulin glargine resulted in a 44.5% greater reduction in TGs compared with pooled TZD treatment [−0.21 *versus* −0.14 mmol/L (−18.5 *versus* −12.8 mg/dL), respectively; *p* = 0.0504]. High-density lipoprotein cholesterol levels increased significantly more with pooled TZD than with insulin glargine therapy (*p* < 0.0001).

**Table 2:** Changes from baseline in lipid levels, pooled analysis

	Unadjusted			Adjusted^a^		
		
	Baseline^b^	Endpoint^b^	Change from baseline (%)	Change from baseline (%)	Difference in change from baseline (%)	*p* value
LDL-C (mg/dL)						
Insulin glargine	109.21 ± 2.1	105.92 ± 2.1	−3.01 ± 1.7	−1.9	−7.9	0.0003
TZD	106.67 ± 2.2	113.47 ± 2.0	6.37 ± 2.0	6.6		
HDL-C (mg/dL)						
Insulin glargine	42.29 ± 1.5	42.98 ± 1.6	1.63 ± 0.9	1.1	−7.6	<0.0001
TZD	41.46 ± 1.4	45.83 ± 1.5	10.52 ± 1.2	9.4		
Non-HDL-C (mg/dL)						
Insulin glargine	151.20 ± 1.7	140.92 ± 1.7	−6.80 ± 1.3	−5.9	−7.5	<0.0001
TZD	150.50 ± 1.7	151.74 ± 1.8	0.82 ± 1.6	1.7		
Total cholesterol (mg/dL)						
Insulin glargine	195.58 ± 1.4	186.20 ± 1.3	−4.80 ± 1.0	−4.2	−7.8	<0.0001
TZD	194.24 ± 1.3	201.10 ± 1.3	3.53 ± 1.3	4.0		
Triglycerides (mg/dL)						
Insulin glargine	194.27 ± 4.2	156.22 ± 4.0	−19.59 ± 2.8	−18.5	−6.5	0.0504
TZD	198.79 ± 3.6	169.64 ± 3.5	−14.66 ± 2.7	−12.8		
Free fatty acids (mg/dL)						
Insulin glargine	52.92 ± 2.7	38.32 ± 3.0	−27.59 ± 3.5	−25.8	−7.3	0.0528
TZD	51.15 ± 2.5	40.89 ± 2.8	−20.05 ± 3.3	−20.0		

Data are mean ± SE.

HDL-C, high-density lipoprotein cholesterol; LDL-C, low-density lipoprotein cholesterol; non-HDL-C, non-high-density lipoprotein cholesterol; TZD, thiazolidinedione.

a Adjusted for study and baseline lipid concentration.

b Geometric means.

In spite of elevated LDL-C, non-HDL-C, and TG levels at baseline, 61.4% of participants in the analysis were not taking lipid-lowering medications. Among these patients, 7.5% in the insulin glargine group and 5.3% in the TZD group were started on statin/fibrate therapy during the study. When patients‘ use of statin/fibrate medication was controlled, the differences for insulin glargine *versus* pooled TZD treatment were unchanged. This analysis also evaluated the lipid effects of insulin glargine and pooled TZD treatment separately for patients who were taking a statin or fibrate and those who were not (Table 3). Insulin glargine produced greater improvement in LDL-C, non-HDL-C, TC, TGs, and HDL-C in patients already taking a lipid-lowering medication compared with those who were not. For the pooled TZD group, an increase in LDL-C was greater in patients taking a lipid-lowering medication, whereas an increase in TC was greater in patients receiving no lipid-lowering treatment. TZD-related improvement in HDL-C and TGs was greater in patients taking a lipid-lowering medication, whereas improvement in FFAs was greater in those not taking the medication.

**Table 3:** Changes in lipid levels by statin/fibrate use, pooled analysis

	Insulin glargine *versus* pooled TZD^a^	Statin/fibrate therapy	No statin/fibrate therapy
			
	*p* value	% Change	% Change
LDL-C	0.0003		
Insulin glargine		−5.51 ± 3.73	−1.55 ± 1.69
TZD		8.10 ± 4.11	5.44 ± 2.08
HDL-C	<0.0001		
Insulin glargine		2.76 ± 1.37	0.90 ± 1.11
TZD		11.67 ± 1.51	9.84 ± 1.68
Non-HDL-C	<0.0001		
Insulin glargine		−9.07 ± 2.62	−5.29 ± 1.33
TZD		−0.97 ± 3.24	1.93 ± 1.67
Total cholesterol	0.0015		
Insulin glargine		−6.30 ± 1.93	−3.80 ± 1.09
TZD		2.44 ± 2.55	4.20 ± 1.32
Triglycerides	0.0641		
Insulin glargine		−23.16 ± 5.01	−17.16 ± 3.41
TZD		−21.11 ± 5.20	−10.50 ± 3.01
Free fatty acids	0.0547		
Insulin glargine		−27.08 ± 5.50	−27.93 ± 4.54
TZD		−18.05 ± 5.99	−21.23 ± 3.89

Data are mean ± SE.

HDL-C, high-density lipoprotein cholesterol; LDL-C, low-density lipoprotein cholesterol; non-HDL-C, non-high-density lipoprotein cholesterol; TZD, thiazolidinedione.

Adjusted for statin/fibrate use and baseline lipid concentration.

Attainment of the recommended lipid goals was assessed at the end of treatment for LDL-C, non-HDL-C, and TGs. Compared with TZDs, insulin glargine treatment enabled more patients to reach the goals for LDL-C (32.7 *versus* 37.2%), non-HDL-C (29.1 *versus* 38.8%), and TGs (43.5 *versus* 49.6%). Patients treated with insulin glargine or pioglitazone generally showed comparable goal attainment, but fewer patients taking rosiglitazone reached the lipid goals. For example, 19% more patients taking insulin glargine than those taking rosiglitazone reached the goals for non-HDL-C and TGs. Compared with insulin glargine, pioglitazone enabled more patients to attain the goals for LDL-C/HDL-C <3.5 (91.5 *versus* 87.3%) and TC/HDL-C <4.5 (66.1 *versus* 55.0%). Overall, however, lipid goal attainment remained suboptimal in most patients.

Baseline lipid measurements were categorized into quartiles to determine whether the changes observed in lipid parameters varied by baseline concentration. Changes from baseline were analysed by baseline quartile and treatment (insulin glargine, rosiglitazone, or pioglitazone) for each lipid parameter (Figure). Improvement in lipid levels differed significantly depending on baseline concentration (*p* < 0.0001 for LDL-C, non-HDL-C, TC, TGs, and FFAs; *p* = 0.0252 for HDL-C), with the greatest improvement generally observed among patients who had the most abnormal lipid concentrations at baseline. For example, treatment with insulin glargine or pioglitazone produced an average decrease in LDL-C of more than 20 mg/dL among patients in the highest quartile at baseline; however, patients in the lower two quartiles experienced a modest increase. Significant treatment differences were obtained for LDL-C (*p* < 0.0001), HDL-C (*p* < 0.0001), non-HDL-C (*p* < 0.0001), TC (*p* < 0.0001), and TGs (*p* = 0.0298). Insulin glargine was better than rosiglitazone in reducing LDL-C (*p* < 0.0001), non-HDL-C (*p* < 0.0001), TC (*p* < 0.0001), and TGs (*p* = 0.0361). Pioglitazone produced greater improvement than did rosiglitazone in LDL-C (*p* < 0.0001), HDL-C (*p* < 0.0001), non-HDL-C (*p* < 0.0001), TC (*p* < 0.0001), and TGs (*p* = 0.0094). Insulin glargine and pioglitazone produced comparable benefits, except for greater reduction in TC with insulin glargine (*p* = 0.0481) and greater improvement in HDL-C with pioglitazone (*p* < 0.0001). Overall, both insulin glargine and pioglitazone had beneficial effects on lipid concentrations; however, rosiglitazone had deleterious effects on LDL-C, non-HDL-C, and TC.

**Figure 1:**
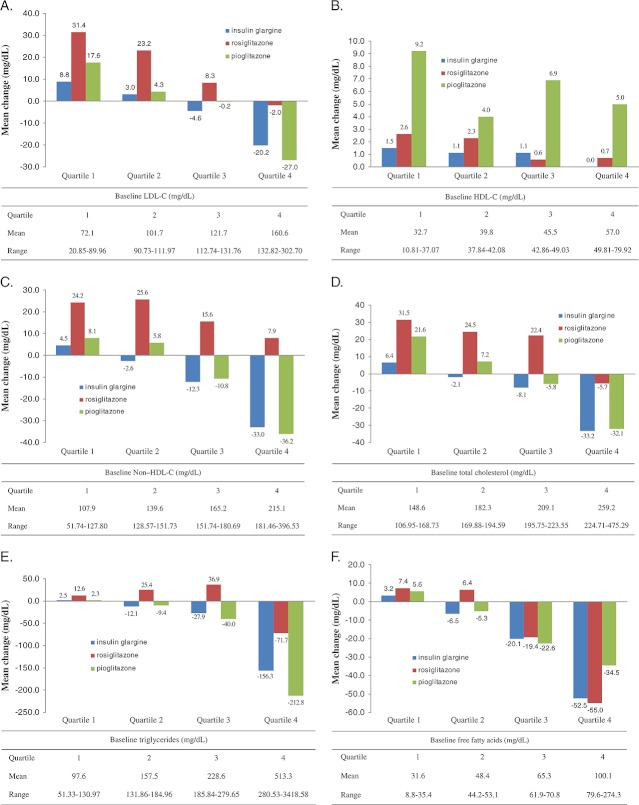
Changes in plasma lipid levels by treatment and baseline lipid quartile. (A) Low-density lipoprotein cholesterol (LDL-C); (B) high-density lipoprotein cholesterol (HDL-C); (C) non-high-density lipoprotein cholesterol (non-HDL-C); (D) total cholesterol; (E) triglycerides; (F) free fatty acids

Using stepwise regression models, we examined various factors (covariates) that could serve as predictors of changes in lipid levels in this pooled analysis. The amount of variability in lipid measures that was explained by each covariate is presented in Table 4. Baseline lipid concentration explained the largest amount of variability for each lipid parameter except HDL-C; however, no other covariate accounted for more than 5% of the variability for any lipid level. Differences between insulin glargine and pooled TZD treatment were significant predictors for LDL-C, HDL-C, non-HDL-C, TC, and TGs. For each lipid parameter except HDL-C, insulin glargine was associated with greater improvement in lipid outcomes. Baseline A1C, baseline weight, change in A1C, and change in weight did not account for the variability in any lipid parameter except FFA.

**Table 4:** Predictors of change in plasma lipid levels per stepwise regression analysis

Outcome	Covariate	Variance explained (%)	*p* value
LDL-C	LDL-C at baseline	28.47	<0.0001
	HDL-C at baseline	2.10	0.0008
	Concomitant statin/fibrate use: no *versus* yes	1.83	0.0003
	Treatment group: TZD *versus* insulin glargine	1.54	0.0008
	Study: rosiglitazone *versus* pioglitazone	1.49	0.0032
	Race: nonwhite *versus* white	0.61	0.0281
	Sex: female *versus* male	0.43	0.0789
HDL-C	Treatment group: TZD *versus* insulin glargine	6.09	<0.0001
	Background use of both SU and metformin: no *versus* yes	4.60	0.0005
	HDL-C at baseline	3.00	<0.0001
	Sex: female *versus* male	1.59	0.0025
	Background SU: no *versus* yes	0.56	0.0458
	Age (years)	0.47	0.0188
	Race: nonwhite *versus* white	0.52	0.0898
Non-HDL-C	Total cholesterol at baseline	25.28	<0.0001
	Study: rosiglitazone *versus* pioglitazone	3.36	<0.0001
	Treatment group: TZD *versus* insulin glargine	2.64	<0.0001
	Concomitant statin/fibrate use: no *versus* yes	1.83	0.0002
	Race: nonwhite *versus* white	1.22	0.0036
Total cholesterol	Total cholesterol at baseline	26.71	<0.0001
	Treatment group: TZD *versus* insulin glargine	4.31	<0.0001
	Study: rosiglitazone *versus* pioglitazone	1.91	0.0014
	Concomitant statin/fibrate use: no *versus* yes	1.57	0.0005
	Race: nonwhite *versus* white	1.05	0.0186
	HDL-C at baseline	0.44	0.0751
Triglycerides	Triglyceride at baseline	28.61	<0.0001
	Study: rosiglitazone *versus* pioglitazone	3.83	<0.0001
	HDL-C at baseline	2.40	0.0001
	Race: nonwhite *versus* white	1.15	0.0036
	Treatment group: TZD *versus* insulin glargine	0.76	0.0200
	Concomitant statin/fibrate use: no *versus* yes	0.47	0.0631
FFA	FFA at baseline	50.93	<0.0001
	A1C at baseline	1.18	0.0092
	Diastolic blood pressure at baseline	0.76	0.0045
	Study: rosiglitazone *versus* pioglitazone	0.78	0.0048
	Sex: female *versus* male	0.48	0.0226
	Treatment group: TZD *versus* insulin glargine	0.35	0.0610

Note: FFA, free fatty acid; HDL-C, high-density lipoprotein cholesterol; LDL-C, low-density lipoprotein cholesterol; non-HDL-C, non-high-density lipoprotein cholesterol; SU, sulphonylurea; TZD, thiazolidinedione.

### Changes in glycaemic control

Both insulin glargine (adjusted mean ± SE Δ = −2.04 ± 0.06%; *p* < 0.0001) and TZDs (adjusted mean ± SE −1.68 ± 0.06%; *p* < 0.0001) reduced A_1C_, with insulin glargine producing a greater reduction in A1C than TZDs (mean ± SE difference = −0.36 ± 0.09%; *p* < 0.0001); however, the difference was not significant for patients with BMI >35 kg/m^2^. Both treatment groups also yielded significant reductions in FPG [insulin glargine: adjusted mean ± SE Δ = −4.51 ± 0.16 mmol/L (−81.33 ± 2.9 mg/dL), *p* < 0.0001; TZD: adjusted mean ± SE Δ = −3.21 ± 0.16 mmol/L (−57.91 ± 2.8 mg/dL), *p* < 0.0001. Improvement in FPG was significantly greater with insulin glargine than with TZD therapy [mean ± SE difference = −1.30 ± 0.22 mmol/L (−23.42 ± 3.9 mg/dL), *p* < 0.0001], although the difference was not significant for patients with BMI >40 kg/m^2^. Fifty percent of patients treated with insulin glargine and 42% of patients in the pooled TZD group achieved A_1C_ ≤7.0% by the end of treatment.

### Safety

Symptomatic hypoglycaemia [defined as symptoms of hypoglycaemia confirmed by a blood glucose level of <3.9 mmol/L (<70 mg/dL)] was reported by 32.6% of patients treated with insulin glargine and 21.9% of patients treated with a TZD. Severe hypoglycaemia [defined as symptomatic hypoglycaemia requiring third-party assistance with a blood glucose level of <2.0 mmol/L (<36 mg/dL) or recovery after oral carbohydrate, intravenous glucose, or glucagon administration] occurred in 2.6% of insulin-glargine-treated and 2.4% of TZD-treated patients. Treatment-related peripheral oedema was reported in 6.6% of TZD-treated and 0% of insulin-glargine-treated patients.

Weight increased in both the insulin glargine (adjusted mean ± SE Δ = 1.83 ± 0.27 kg) and TZD (adjusted mean ± SE Δ = 2.98 ± 0.26 kg) groups; however, TZD produced significantly greater weight gain (mean ± SE difference = 1.14 ± 0.37 kg; *p* = 0.0024, compared with glargine). There was a progressive increase in weight gain across the BMI groups for TZD but not for insulin glargine. The largest difference in weight gain was observed in patients with BMI ≥40 kg/m^2^ (adjusted mean ± SE difference = −2.40 ± 1.10 kg; *p* = 0.0324).

## Discussion

Whereas the effects of TZD on lipids are well known, the effects of insulin on lipids are not generally appreciated; in fact, insulin therapy has been hypothesized to have deleterious effects on lipids in patients with type 2 diabetes [8–10]. Our analysis shows that insulin glargine treatment results in greater improvement in lipid levels and more patients reaching the ADA/AHA recommended lipid goals compared with pooled TZD treatment in combination with metformin with or without sulphonylureas. The beneficial effects of insulin glargine are greater in patients on lipid-lowering therapy. Generally, the lipid benefits of insulin glargine are comparable with those of pioglitazone and better than those of rosiglitazone. Reductions in A1C and FPG and achievement of A_1C_ ≤7.0% are greater than those observed with pooled TZD treatment, especially for less obese patients. As expected, hypoglycaemia was more common among patients treated with insulin glargine than among those treated with a TZD, but episodes of severe hypoglycaemia were low in both treatment groups.

Previous studies have shown an improvement in lipid profiles of diabetic patients treated with insulin [17,22,23]. Treatment with 70% NPH insulin/30% regular insulin has been shown to reduce TC and TGs in patients with type 2 diabetes [17]. Twenty-four weeks of insulin glargine with standard oral anti-diabetic drug therapy decreases TC, non-HDL-C, and TGs [15]. Insulin glargine or NPH insulin combined with metformin results in a 27% to 29% decrease in TGs and a 5% to 6% increase in HDL-C [18]. The current analysis extends the information in the literature regarding the effects of insulin glargine on all lipid parameters, as well as the lipid ratios and lipid goals achieved in patients with type 2 diabetes.

The effect of TZDs on lipids in this analysis is generally consistent with previous reports on TZDs in patients with type 2 diabetes. In a meta-analysis and in previous studies, pioglitazone has been shown to reduce TGs and LDL-C/HDL-C and increase HDL-C levels [24,25]. Rosiglitazone improves HDL-C; however, its effects on LDL-C and TC are variable [26]. In a prospective randomized clinical study comparing pioglitazone and rosiglitazone in the treatment of patients with type 2 diabetes and dyslipidaemia, significantly better outcomes in TG, HDL-C, and LDL-C levels were reported for pioglitazone than for rosiglitazone [27].

Given the evidence that statins improve clinical outcomes and the large number of patients with elevated LDL-C, non-HDL-C, and TGs at baseline, it was surprising that more than 60% of patients were not receiving lipid-lowering therapy. It is of interest that insulin glargine produced greater improvement in lipid levels in patients on statins or fibrates, and a greater number of patients on insulin glargine attained the ADA/AHA goals for LDL-C, non-HDL-C, and TGs in this study. It is encouraging that the greatest reduction in LDL-C occurred in patients with the highest baseline levels. However, the finding of increased LDL-C among patients in the lower baseline quartiles reinforces the importance of monitoring patients with elevated risk for cardiovascular disease and providing lipid-lowering therapy when appropriate.

Insulin regulates FFA, TG, and lipoprotein particle metabolism through a variety of mechanisms, which may account for the observed beneficial lipid effects of insulin glargine in this analysis. Insulin suppresses lipolysis, preventing the release of FFAs from adipose tissue [28], and increases the plasma clearance of FFAs [29]; these effects result in a reduction of available substrate for hepatic TG production [28]. Insulin also suppresses the hepatocyte production of TGs and very low density lipoproteins *in vitro* [30,31] and *in vivo* [29,32]. Moreover, insulin is a potent activator of lipoprotein lipase, which enhances the catabolism of TG-rich lipoproteins [28]. A significant decrease in hepatic lipase activity also has been observed following insulin therapy, which may reduce the production of highly atherogenic small dense LDL particles [23]. Insulin may promote LDL-C clearance by enhancing LDL degradation [33,34]. Finally, insulin regulates HDL particle synthesis through induction of apolipoprotein A-1 gene expression in the liver [35].

Hyperglycaemia and dyslipidaemia are associated with an increased risk of cardiovascular events in patients with type 2 diabetes [36,37]. Our analysis suggests that insulin has beneficial effects on lipids and glucose in patients with type 2 diabetes. The clinical impact of this observation on cardiovascular risk reduction will be addressed specifically in the ORIGIN (Outcome Reduction with Initial Glargine Intervention) trial. This randomized controlled study was designed to evaluate the effects of insulin glargine on cardiovascular risk and mortality in a variety of dysglycaemic patients (i.e. impaired fasting glucose, impaired glucose tolerance, diabetes) who are at high risk for a cardiovascular event [38]. Therefore, the findings of this study will be applicable to a broad spectrum of patients with glycaemic abnormalities [38].

The choice of antihyperglycaemics should be individualized according to the patient's medical history, disease characteristics, comorbidities, baseline A_1c_, lifestyle, body weight, and risk of adverse events. Insulin glargine might be more appropriate for patients requiring a greater reduction in A_1c_, whereas TZD might be more appropriate for patients with a history of severe hypoglycaemia. On the basis of our study, either insulin glargine or pioglitazone can be chosen if additional benefits on lipids are a consideration for choosing an antihyperglycaemic agent. Insulin glargine might be more appropriate, for instance, in patients taking a lipid-lowering medication, in light of the synergistic effects of statins and insulin glargine observed in this study, whereas pioglitazone may be preferred if HDL is very low. Either insulin glargine or pioglitazone may be beneficial in patients at risk for cardiovascular disease.

### Analysis limitations

This analysis pooled results from studies of rosiglitazone and pioglitazone [19,20], which are known to have different effects on lipid parameters [39,40]. The lipid results, therefore, are presented separately for rosiglitazone and pioglitazone. Glycaemic outcomes are presented only for pooled TZD treatment because TZDs have similar glycaemic effects [39,40]. Differences attributable to other anti-diabetic agents cannot be evaluated, as participants in the rosiglitazone study received both metformin and a sulphonylurea [19] whereas participants in the pioglitazone study received either one or the other [20]. However, TZD use accounted for variability only in HDL-C in the lipid regression models. Furthermore, doses of statin and fibrate medications were not held constant during treatment. The possibility that potential dosage titrations of these lipid-lowering medications contributed to the observed lipid effects cannot be excluded. A future comparative study of insulin glargine *versus* pioglitazone, with the addition of more detailed lipid measurements, such as apolipoproteins and small dense LDL particles, and with statin dose held constant during the treatment period, would provide further insight into the lipid-lowering effects of these two medications.

### Safety

The higher frequency of episodes of hypoglycaemia with insulin glargine and weight gain and peripheral oedema with TZD in our analysis are consistent with the known side effects of these agents. Episodes of severe hypoglycaemia occurred in fewer than 3% of patients receiving either treatment [19,20].

## Conclusion

Treatment with insulin glargine showed beneficial effects on lipid parameters, which are generally comparable with the effects of pioglitazone and more favourable than those of rosiglitazone. Lipid improvement occurred within the context of better glycaemic control attained with insulin glargine. Insulin glargine treatment led to more hypoglycaemia but less weight gain and oedema compared with TZD treatment. Both insulin glargine and TZDs promoted changes in lipid profile; however, there were some specific qualitative and quantitative differences depending on the agent used to lower blood glucose. These findings suggest that favourable effects on plasma lipid profiles should be considered among the advantages of treatment with insulin glargine as they are for TZDs.
